# Genetics of IL6 polymorphisms: Case–control study of the risk of endometrial cancer

**DOI:** 10.1002/mgg3.600

**Published:** 2019-03-03

**Authors:** Junhong Cai, Kaiying Cui, Fanglin Niu, Tianbo Jin, Sizhe Huang, Ying Zhang, Shan Bao

**Affiliations:** ^1^ Key Laboratory of Cell and Molecular Genetic Translational Medicine in Hainan Province Hainan China; ^2^ Department of Gynaecology and Obstetrics Hainan General Hospital Hainan China; ^3^ Key Laboratory of Resource Biology and Biotechnology in Western China (Northwest University) Ministry of Education Xi’an, Shaanxi China; ^4^ School of Life Sciences Northwest University Xi’an, Shaanxi China

**Keywords:** Case–control study, endometrial cancer, genetic polymorphisms, IL6

## Abstract

**Background:**

Endometrial cancer is the most common gynaecological malignancy. Cytokines gene may be important in endometrial cancer development. This study sought to investigate whether the *IL4*, *IL6* two gene genetic variants were associated with susceptibility to endometrial cancer (EC) in Hainan Chinese Han women by a hospital‐based study.

**Methods:**

The genetic polymorphisms for *IL4* and *IL6* were analyzed by Agena MassARRAY method. Odds ratios (ORs) and 95% confidence intervals (CIs) were calculated by unconditional logistic regression.

**Results:**

We observed a significant increase in risk of endometrial cancer of rs1524107 (*IL6*) (T/C, OR = 1.61, 95% CI = 1.09–2.37, *p* = 1.55 × 10^−2^), rs2066992 (*IL 6*) (OR = 3.09, 95% CI = 2.11–4.53, *p* = 3.13 × 10^−9^). However, for *IL4* gene, no associations emerged the SNP and EC risk.

**Conclusion:**

This study demonstrated that *IL6* gene polymorphisms are significantly associated with increased EC susceptibility in Hainan Chinese Han women.

## 1 INTRODUCTION

Endometrial cancer (EC) is the second most common female genital tract carcinoma in world (N & DS, 2009). The number of endometrial cancer cases has been increasing worldwide and the incidence varies among regions and races (Sorosky, 2012). More than 287,100 new EC cases are diagnosed each year, and the mortality rate of EC has increased significantly during the past two decades (Jemal et al., 2011; Sorosky, 2012). Identifying populations at risk for endometrial cancer is of particular importance for cancer prevention.

The etiology of the endometrial carcinoma is not fully understood. Several well‐established endometrial cancer risk factors such as, age, hyperestrogenism, obesity, anovulation, the history of sterility, low parity, and late menopause (Busch et al., 2017; Gao et al., 2015; Ghanbari, Agajani, Moslemi, & Esmaeilzadeh, 2016). Many of these risk factors may also exert proinflammatory carcinogenic effects. In recent years, the relationship between inflammation and cancer has become a hot issue of cancer researches. Therefore, single nucleotide polymorphisms (SNPs) in genes involved in encoding inflammation response molecules may affect. Lots of studies have provided evidences: elevated levels of several inflammatory markers such as C‐reactive protein (CRP) (Wen et al., 2008), interleukin 6 (IL‐6) (Che, Liu, Wang et al., 2014), and IL1receptor antagonist (IL1Ra) significantly increased the risk of endometrial cancer (Yu et al., 2016). Although large numbers of studies have documented a relationship between inflammation and EC development, the present understanding of the mechanisms of EC is still inadequate. As mentioned above, to the best of our knowledge, this is the first study to describe the association between *IL4, IL6* gene polymorphisms and susceptibility to endometrial cancer in Hainan women.

## 2 MATERIALS AND METHODS

### 2.1 Subjects

The study included 81 newly diagnosed endometrial cancer cases (aged 54.09 ± 10.78 years) recruited from Hainan General Hospital. The clinical stage of EC was defined according to the International Federation of Gynecology and Obstetrics (FIGO, 2014) criteria 198 population controls (aged 54.08 ± 9.68 years) were accrued from healthy volunteers who visited the hospitals between July 2016 and July 2017 for general health exams. Controls had no history of hysterectomy, endometrial ablation, or previous cancer and were frequency age‐matched. In‐person interviews were conducted with participants by retired medical professionals to gather information on demographics, dietary intake, lifestyle factors, disease history, family history of any cancer, menstrual and reproductive history, and hormone use. All subjects were unrelated ethnic Han Chinese. The written informed consent was obtained from each participant. The study was approved by the Hainan General Hospital. Each study participant provided 5 ml peripheral blood sample.

### 2.2 Selection of tag SNPs and genotyping

In this study, 11 SNPs that had minor allele frequencies (MAF) exceeding 5% in *IL4* and *IL6* were selected from DbSNP database (http://www.hapmap.org/index.html.en) and SNP Consortium database (http://snp.cshl.org/) for analysis. DNA was isolated from Whole blood was used the GoldMag‐Mini Whole Blood Genomic DNA Purification Kit (GoldMag Co. Ltd. Xi'an City, China) extracted. Genotypes for SNPs were determined by Agena MassARRAY. We used a NanoDrop 2000 (Gene Company Limited) was measured DNA concentrations. We used Agena MassARRAY Assay Design 3.0 Software to design a Multiplexed SNP MassEXTEND assay (Gabriel, Ziaugra, & Tabbaa, 2009; Thomas et al., 2007). The PCR primers for each SNP are shown in Table 1. Data management and analysis was performed using the Agena Typer 4.0 Software (Thomas et al., 2007).

**Table 1:** PCR primers

SNP	1st‐PCR primer sequences	2st‐PCR primer sequences	UEP sequences
rs2243250	ACGTTGGATGTAACAGGCAGACTCTCCTAC	ACGTTGGATGTGATACGACCTGTCCTTCTC	TAAACTTGGGAGAACATTGT
rs2227284	ACGTTGGATGGATGAAGGGTTTCTTGGGTG	ACGTTGGATGCATTATGGAACTCTCTGTAG	AGCTCTCTTTGGTAAATAGGAAAT
rs2243267	ACGTTGGATGTATAGTTTACTCACTGCCGC	ACGTTGGATGAGAAACGCATTGCACAGTGG	cccaCTATCGTGGCAGATTTTTG
rs2243270	ACGTTGGATGCAGTATCAACAGTTGACCCC	ACGTTGGATGACATTCACTCATCCCACCAG	CACCAGCCAGAGGTAACTA
rs2243283	ACGTTGGATGAAACAGTACTGACCATCGCC	ACGTTGGATGTGCTGACAGATCGGTTGTAG	tGGGGAGGAAAAGATGAC
rs2243289	ACGTTGGATGGGCTTGATCAAGTAGACAGG	ACGTTGGATGTCACAGGACAGGAATTCTGC	tatCTTGCATTGGTAAGCATTTGTC
rs1800796	ACGTTGGATGTCTTCTGTGTTCTGGCTCTC	ACGTTGGATGTGGAGACGCCTTGAAGTAAC	ccccGCAGTTCTACAACAGCC
rs2069837	ACGTTGGATGTCTCCAAAAACCTTCCTTGC	ACGTTGGATGCTGCTGGAACATTCTATGGC	GTGTGCCAGGCACTTTA
rs1524107	ACGTTGGATGGGATGCCAATGAGTTGTAGC	ACGTTGGATGAACAAACACCACTAGAGGGC	gAACCACAGCCAGGAAA
rs2066992	ACGTTGGATGTAGAGACTTTCCTGGCTGTG	ACGTTGGATGTGTTATCACCTAAGTGTCCC	ccacCAAACACCACTAGAGGG
rs2069840	ACGTTGGATGCCAGGCAGCAACAAAAAGTG	ACGTTGGATGCTGTCCAAGAATAAACTGCC	caTGCCTTTAAAAAAGCTTGAA

### 2.3 Statistical analysis

For each polymorphism, deviation of the genotype frequencies in the controls from those expected under Hardy–Weinberg equilibrium was assessed using the standard χ^2^‐test. Genotype frequencies in cases and controls were compared by χ^2^‐tests. The adjusted odds ratios (ORs) and 95% confidence intervals (CIs) for the effect of genotype on the risk of EC were estimated by the logistic regression analysis. Tests for the associations of each SNP and haplotype with EC were estimated by using the Haploview software *p*‐values<0.05 were considered to be significant.

## 3 RESULTS

In this study, the genotypes of 279 samples were determined. Eighty‐one cases and 198 controls were in our study. The control and case age mean was 54.08 ± 9.68, 54.09 ± 10.78, respectively and the age distributions were similar for cases and controls (*p* = 0.921).Table 2 shows the distributions of the genotypes and alleles of the *IL‐4*, *IL6 *polymorphisms. The genotype distributions for each SNP were consistent with Hardy‐Weinberg equilibrium (HWE).The rs1524107 (T/C) of *IL6* imposed significant 1.61‐fold risk for EC patients (OR = 1.61, 95% CI = 1.09–2.37, *p* = 1.55 × 10^−2^). The rs2066992 (T/G) in *IL6* gene was associated with EC risk (OR = 3.09, 95% CI = 2.11–4.53, *p* = 3.13 × 10^−9^).

**Table 2:** Basic information of candidate SNPs in this study

SNP	Chr	Allele(A^a^/B)	Gene	MAF(case)	MAF(control)	P‐HWE	OR	95% CI	*p*
rs2243250	5q31.1	C/T	IL4	0.216	0.253	0.352	0.82	0.53–1.26	0.361
rs2227284	5q31.1	G/T	IL4	0.148	0.169	0.803	0.85	0.51–1.42	0.541
rs2243267	5q31.1	G/C	IL4	0.222	0.250	0.569	0.86	0.55–1.32	0.487
rs2243270	5q31.1	A/G	IL4	0.222	0.250	0.569	0.86	0.55–1.32	0.487
rs2243283	5q31.1	G/C	IL4	0.142	0.172	0.612	0.8	0.48–1.33	0.388
rs2243289	5q31.1	A/G	IL4	0.216	0.240	0.330	0.87	0.56–1.36	0.545
rs1800796	7p15.3	G/C	IL6	0.346	0.270	0.369	1.43	0.96–2.11	0.075
rs2069837	7p15.3	G/A	IL6	0.179	0.182	0.098	0.98	0.61–1.58	0.938
rs1524107	7p15.3	T/C	IL6	0.381	0.277	0.049	1.61	1.09–2.37	1.55E−02
rs2066992	7p15.3	T/G	IL6	0.531	0.268	0.148	3.09	2.11–4.53	3.13E−09
rs2069840	7p15.3	G/C	IL6	0.130	0.081	0.118	1.69	0.94–3.04	0.074

Note

MAF, minor allelic frequency; HWE, Hardy–Weinberg Equilibrium; ORs, odds ratios; CI: confidence interval.

HWE *p*‐value ≤0.05 was excluded; *p* value ≤0.05 indicates statistical significance.

a Minor allele.

Furthermore, we assumed that the minor allele of each SNP as a risk factor compared with the wild‐type allele. Four genetic models (codominant, dominant, recessive, and log‐additive) were applied to analyze the SNP and EC association analysis with adjustments for age in Table 3. Significantly increased risk of EC was found to be associated with the CC genotype of rs1524107 in the recessive model, compared with CT/TT genotype (OR = 2.26, 95% CI = 1.12–4.56, *p* = 0.024) using age adjustment. The rs2066840 genotypic OR for the homozygous genotype (GG) was 9.85 (5.10–19.00, *p* < 0.05) compatible with recessive genetic models. In codominant model the heterozygous genotype (GT) was decreased likelihood of EC and homozygous genotype (GG) was increased likelihood of EC (OR = 0.27, 95% CI = 0.11–0.69, *p* < 0.05; OR = 6.97, 95% CI = 3.53–13.78, *p* < 0.05, respectively). No significant difference was observed in the other SNPs between the two study groups.

**Table 3:** Association between the SNPs and risk of EC in genetics models

SNP	Model	Genotype	Control	Case	OR^a^ (95% CI)	P^a^‐value	OR^b^ (95% CI)	P^b^‐value
rs1524107	Codominant	T/T	109 (55.3%)	36 (45%)	1.00	0.06	1.00	0.06
		C/T	67 (34%)	27 (33.8%)	1.22 (0.68–2.19)		1.24 (0.69–2.24)	
		C/C	21 (10.7%)	17 (21.2%)	2.45 (1.17–5.15)		2.50 (1.18–5.31)	
	Dominant	T/T	109 (55.3%)	36 (45%)	1.00	0.12	1.00	0.11
		C/T‐C/C	88 (44.7%)	44 (55%)	1.51 (0.90–2.55)		1.54 (0.90–2.61)	
	Recessive	T/T‐C/T	176 (89.3%)	63 (78.8%)	1.00	0.03	1.00	0.02
		C/C	21 (10.7%)	17 (21.2%)	2.26 (1.12–4.56)		2.29 (1.13–4.63)	
	Log‐additive	—	—	—	1.49 (1.04–2.13)	0.03	1.51 (1.05–2.17)	0.03
rs2066992	Codominant	T/T	109 (55.6%)	35 (43.2%)	1.00	<0.0001	1.00	<0.0001
		G/T	69 (35.2%)	6 (7.4%)	0.27 (0.11–0.68)		0.27 (0.11–0.69)	
		G/G	18 (9.2%)	40 (49.4%)	6.92 (3.53–13.58)		6.97 (3.53–13.78)	
	Dominant	T/T	109 (55.6%)	35 (43.2%)	1.00	0.06	1.00	0.06
		G/T‐G/G	87 (44.4%)	46 (56.8%)	1.65 (0.98–2.78)		1.67 (0.98–2.84)	
	Recessive	T/T‐G/T	178 (90.8%)	41 (50.6%)	1.00	<0.0001	1.00	<0.0001
		G/G	18 (9.2%)	40 (49.4%)	9.65 (5.03–18.51)		9.85 (5.10–19.00)	
	Log‐additive	—	—	—	2.27 (1.63–3.17)	<0.0001	2.32 (1.65–3.25)	<0.0001
rs2069840	Codominant	C/C	169 (85.3%)	61 (75.3%)	1.00	0.12	1.00	0.12
		G/C	26 (13.1%)	19 (23.5%)	2.02 (1.05–3.92)		2.03 (1.05–3.94)	
		G/G	3 (1.5%)	1 (1.2%)	0.92 (0.09–9.05)		0.94 (0.09–9.33)	
	Dominant	C/C	169 (85.3%)	61 (75.3%)	1.00	0.05	1.00	0.05
		G/C‐G/G	29 (14.7%)	20 (24.7%)	1.91 (1.01–3.63)		1.92 (1.01–3.66)	
	Recessive	C/C‐G/C	195 (98.5%)	80 (98.8%)	1.00	0.86	1.00	0.85
		G/G	3 (1.5%)	1 (1.2%)	0.81 (0.08–7.93)		0.81 (0.08–8.00)	
	Log‐additive	—	—	—	1.65 (0.93–2.91)	0.09	1.66 (0.93–2.95)	0.09

Note

OR: odds ratio; 95% CI: 95% confidence interval.

*p* < 0.05 indicates statistical significance.

a Were calculated from two‐sided chi‐square tests or Fisher's exact tests for either genotype distribution.

b Were calculated by unconditional logistic regression adjusted for age.

**Figure 1:**
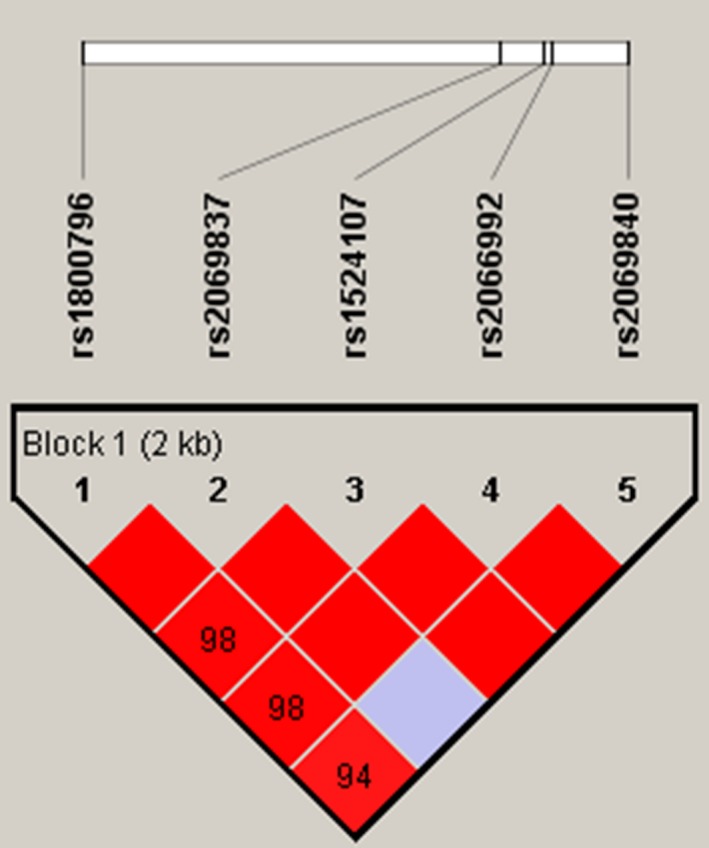
Haplotype block map for the IL6 SNPs genotyped in this study

To determine the extent of LD in the *IL4*, *IL6* gene, genotype data of control groups were used to estimate intermarker LD. Standardized pairwise LD coefficients D and *r* ^2^ between markers were estimated(Figure1). There were no significant differences in the estimated frequencies of these haplotypes between EC patients and controls.

## 4 DISCUSSION

As mentioned above, in our study, we found that the association between *IL6* gene polymorphisms and susceptibility to endometrial cancer in Chinese Han women. In conclusion, the rs1524107 (T/C) and the rs2066992 (T/G) in *IL6* gene seem to be relevant to increased susceptibility to endometrial cancer, which suggests *IL6* may play a role in EC, however, in *IL4* gene, we did not found any SNPs were imposed significant with EC risk.

The research found the three proinflammatory cytokines interleukin (IL)‐1, IL‐6 and tumor necrosis factor (TNF)‐alpha, are all involved in the development of endometriosis (Cheong et al., 2002). In rats, increased serum IL‐6 levels after surgical induction of endometriosis suggest that IL‐6 may be involved in the initial development of endometriosis (Lim & Schenken, 1993). IL‐6 regulates immune and inflammatory responses in physiological conditions, but recent reports suggest that IL‐6 expression is implicatedin the regulation of tumor growth and metastatic spread, including breast cancer and other gynecological tumors (Drygin et al., 2011; Sidhu, Miller, & Hollenbach, 2011). The previous study proved that IL‐6 localized in endometrial cancer cells and promoted cancer progression via a paracrine manner and demonstrated that IL‐6 activation was associated with endometrial cancer development by inducing aromatase expression in intratumoral stromal cells (Che, Liu, Liao et al., 2014). In the normal uterine epithelium, IL‐6 is known to promote the proliferation, invasion and differentiation of trophoblast cells (Salgado et al., [Link]). Trophoblast cells of the developing embryo actively secrete IL‐6 (MJ et al.., 2010). IL‐6 is also involved in the promotion of neoplastic change. IL‐6 also has a known growth‐promoting role in tumor growth and metastasis IL‐6 has also been implicated in the progression of uterine malignancies (Darai, Detchev, & Quang, 2003; Ferdeghini et al., 1994).

In this study, we investigated rs1800796, rs2069837, rs1524107, rs2066992，rs2069840 five SNPs in *IL6*, and six SNPs in *IL4*, including the rs2243250, rs2227284, rs2243267, rs2243270, rs2243283, and rs2243289. Among these SNPs, rs1524107, and rs2066992 were identified that have association with EC risk. Rs1524107 was identified that has associations with several disease, including rheumatoid arthritis (Li et al., 2014), major depressive disorder (Chen, Wu, Zhao, Fan, & Fang, 2016), Alzheimer's disease (Chen et al., 2012), adult‐onset asthma (Lajunen, Jaakkola, & Jaakkola, 2016), lung cancer (Chen et al., 2013) et al, however, we did not found any reports association with EC risk in Chinese han population. For rs2066992, Juo et al., 2009 researched the rs2066992 and endometriosis risk, however, there is no significance. Other studies have found rs2066992 were found associated with CAD (Ding et al., 2015), chronic hepatitis B virus infection (Zhao, Gao, Zhou, Pan, & Li, 2013), antipsychotic (Fonseka et al., 2015).

Although we detected the association between the SNPs in *IL4*, *IL6* and EC, there were limitations in our study. Firstly, the sample size, especially the sub‐group of different clinical features of EC patients, which was relatively small, might not be large enough to detect the positive effect if it is not strong enough. Further large‐scale studies in diverse ethnic populations are needed to give stronger evidence for this association. Secondly, we were unable to get the information for more environmental factors and lifestyles of the enrolled subjects, which might have an influence on cancer risk. Thirdly, we did not test the expression level of IL4, IL6, which restricted our further research on clarifying the SNPs’ effect on the IL4, IL6 expression level.

In conclusion, this study is novel in demonstrating the association between *IL4*, *IL6* gene polymorphisms and susceptibility to endometrial cancer in Chinese Han women. The study suggests *IL6* may play a role in the process of endometrium carcinogenesis (leading to endometrial cancer). Nevertheless, further studies in different population and with a larger size of samples are necessary to confirm these findings.

## CONFLICT OF INTERESTS

The authors declare that they have no conflict of interest.
